# EXAMINING THE ROLE OF ADVERSITY AND POSITIVE LIFE EVENTS IN THE RELATION BETWEEN GRATITUDE AND WELL-BEING

**DOI:** 10.1093/geroni/igz038.3378

**Published:** 2019-11-08

**Authors:** Omar E Staben, Frank Infurna, Kevin Grimm, Suniya Luthar

**Affiliations:** 1 Arizona State University, Tempe, Arizona, United States; 2 Arizona State University, Tempe, United States

## Abstract

Character strengths are emerging as a key outcome of interest in midlife and old age. One key avenue that has been largely unexplored is what the key antecedents are and the moderating role of adversity and positive life events experiences. The limited current research on the topic has examined the direct relations among character strengths and well-being, whereas less is known regarding the role of negative and positive experiences, which may provide a better understanding of what contributes to character strengths. This study explores whether major life adversities (i.e. personal, family, work related) and positive life events (i.e. job promotion, engagement, vacations) experiences are associated with character strengths— namely gratitude, and well-being. We use data from a sample of participants in midlife (n=362, ages 50-65) who completed monthly online surveys for a period of two years. Multilevel models showed that greater adversity was associated with poorer well-being, whereas positive life events were predictive of higher overall well-being. Individuals’ experience of fewer positive life events was associated with stronger increases in well-being when individuals expressed more gratitude. Conversely, adversity was associated with increasing well-being when individuals expressed more gratitude. Collectively, our findings provide evidence for the role of adversity and positive life experiences to the extent that character strengths have the potential to shape the course of development in adulthood. Our discussion focuses on the potential links that underlie our findings and how they can inform interventions aimed at mitigating the consequences of adversity.

